# MicroRNA expression changes in association with changes in interleukin-1ß/interleukin10 ratios produced by monocytes in autism spectrum disorders: their association with neuropsychiatric symptoms and comorbid conditions (observational study)

**DOI:** 10.1186/s12974-017-1003-6

**Published:** 2017-11-25

**Authors:** Harumi Jyonouchi, Lee Geng, Deanna L. Streck, James J. Dermody, Gokce A. Toruner

**Affiliations:** 1 Department of Pediatrics, Saint Peter’s University Hospital (SPUH), Rutgers-Robert Wood Johnson Medical School, 254 Easton Ave., New Brunswick, NJ 08901 USA; 2 Institute of Genomic Medicine, Rutgers-New Jersey Medical School (NJMS), Newark, NJ USA; 3 Clinical cytogenetics/Department of Hematopathology, MD Anderson Cancer Center, Houston, TX USA

**Keywords:** Autism spectrum disorder (ASD), MicroRNA (miRNA), Interleukin-1ß (IL-1ß), Interleukin-10 (IL-10), Monocytes, Neuroinflammation

## Abstract

**Background:**

MicroRNAs (miRNAs) play a major role in regulating immune responses at post-transcriptional levels. Previously, we have reported fluctuating interlukine-1ß (IL-1ß)/IL-10 ratios produced by peripheral blood monocytes (PBMo) in some patients with autism spectrum disorders (ASD). This study examined whether changes in miRNA expression by PBMo are associated with changes in IL-1ß/IL-10 ratios and how such changes are associated with ASD clinical features.

**Methods:**

miRNA expression by purified PBMo from ASD subjects (*N* = 69) and non-ASD controls (*N* = 27) were determined by high-throughput sequencing. Cytokine production by PBMo in responses to stimuli of innate immunity, and behavioral symptoms [assessed by aberrant behavioral checklist (ABC)] were also evaluated at the same time of sample obtainment.

**Results:**

As a whole, there was no difference in miRNA expression between ASD and control non-ASD PBMo. However, when ASD cells were subdivided into 3 groups with high, normal, or low IL-1ß/IL-10 ratios as defined in the “Results” section, in comparison with the data obtained from non-ASD controls, we observed marked changes in miRNA expression. Namely, over 3-fold changes in expression of miR-181a, miR-93, miR-223, miR-342, and miR-1248 were observed in ASD PBMo with high or low IL-1ß/IL-10 ratios, but not in ASD PBMo with normal ratios. These miRNAs that had altered in expression are those closely associated with the regulation of key signaling pathways. With changes in IL-1ß/IL-10 ratios, we also observed changes in the production of cytokines (IL-6, TNF-α, and TGF-ß) other than IL-1ß/IL-10 by ASD PBMo. The association between behavioral symptoms and cytokine levels was different when ASD cells exhibit high/low IL-1ß/IL-10 ratios vs. when ASD cells exhibited normal ratios. Non-IgE-mediated food allergy was also observed at higher frequency in ASD subjects with high/low IL-1ß/IL-10 ratios than with normal ratios.

**Conclusions:**

Changes in cytokine profiles and miRNA expression by PBMo appear to be associated with changes in ASD behavioral symptoms. miRNAs that are altered in expression in ASD PBMo with high/low IL-1ß/IL-10 ratios are those associated with inflammatory responses. Changes in IL-1ß/IL-10 ratios along with changes in miRNA expression may serve as biomarkers for immune-mediated inflammation in ASD.

**Electronic supplementary material:**

The online version of this article (10.1186/s12974-017-1003-6) contains supplementary material, which is available to authorized users.

## Background

Autism spectrum disorder (ASD) is a behaviorally defined syndrome, encompassing markedly heterogeneous subjects. In addition to their core behavioral symptoms, ASD patients frequently suffer from various co-morbid conditions. GI symptoms and sleep disorders have been most frequently described [1, 2]. The presence of co-morbid conditions can affect ASD behavioral symptoms, partly through pain and discomfort [3, 4]. Therefore, it is difficult to separate the effects of co-morbid conditions on their behaviors from ASD core behavioral symptoms, unless their symptoms are carefully monitored over time with concurrent optimal management of co-morbid conditions.

The presence of multiple co-morbid inflammatory conditions affecting organs other than the brain may be a clue in understanding the versatile clinical features exhibited by some ASD subjects. Since most inflammatory conditions are mediated by the immune system, such ASD patients as described above may have underlying immune-mediated inflammation, affecting both the brain and other organs. In fact, a role of immune-mediated inflammation has been implicated with the onset and progress of ASD by multiple researchers [5, 6]. However, it has been difficult to elucidate how the immune system plays a role in ASD pathogenesis, since there are many immune abnormalities reported in ASD, affecting almost every arm of the immune system [6, 7].

In this regard, results from animal models of autism have provided important clues. The best studied animal model of autism is the maternal immune activation (MIA) model [8]. In this model, innate immune activation via sterile stimulants, such as endotoxin, during a critical period of pregnancy, cause changes in the brain and development of ASD like behaviors in offspring [8]. These results indicate a role of innate immunity in some ASD patients. We have also reported fluctuating changes of innate immune responses in a subset of ASD children, most notable in levels of interleukin (IL)-1ß and IL-10 produced by peripheral blood monocytes [9]. Recently, an animal model of idiopathic autism was developed through conditional mutation of phosphatase and tension homolog (*PTEN*), causing germline mislocalization of *PTEN* [10]. In this model, the authors report progressive disruption of neural gene expression, affecting both the immune and the synaptic pathways over a time, resulting in histological evidence of neuro-inflammation [10]. Apart from the brain, PTEN protein has been shown to exert a crucial role in coordinating phosphatase activities, affecting differentiation and function of regulatory T (Treg) cell and mitochondrial fitness [11]. In humans, *PTEN* mutation could lead to auto-inflammatory and autoimmune symptoms through over-activation of T cells, in addition to impaired immune responses [12]. Interestingly, PTEN expression is regulated by innate immune responses partly through regulation of microRNA (miRNA) expression. For example, miR-181a is found to regulate T cell activation by upregulating PTEN [13].

In this study, we hypothesized that changes in cytokine expression profiles by peripheral blood monocytes (PBMo), especially changes in IL-1ß/IL-10 ratios, are associated with changes in miRNA expression, possibly affecting PTEN-regulated signaling pathways in monocytes, as shown in T cells. In addition, we also hypothesized that changes in such cytokine profiles and miRNA expression are closely associated with behavioral symptoms and co-morbid inflammatory conditions in ASD subjects who reveal fluctuating changes in cytokine production by monocytes (especially IL-1ß and IL-10). To test our hypotheses, in ASD subjects, we studied monocyte cytokine profiles, miRNA expression, and behavioral symptoms assessed at the time of sample obtainment. In those with fluctuating behavioral symptoms, the above described parameters were assessed at multiple data points, if possible. Our results support our hypotheses that changes in miRNA expression parallels to changes in IL-1ß/IL-10 ratios in ASD subjects. Such changes were also associated with changes in associations between behavioral symptoms and monocyte cytokine levels.

## Methods

### Study subjects

The study followed the protocols approved by the Institutional Review Board at our institution Saint Peter’s University Hospital (SPUH), New Brunswick, NJ, USA.

#### *ASD* subjects

ASD subjects were recruited in the Pediatric Allergy/Immunology Clinic. Diagnosis of ASD in the study subjects was made at various autism diagnostic centers, including ours. The ASD diagnosis was based on the Autism Diagnostic Observation Scale (ADOS) and/or Autism Diagnostic Interview-Revisited (ADI-R) and other standard measures. For those who lack verification of ASD diagnosis, the ADOS and/or ADI-R were administered to confirm the diagnosis. Any subjects with deafness/blindness; any motor disability, such as cerebral palsy; or medical conditions with known gene mutations were excluded from the study. ASD subjects were also evaluated for their behavioral symptoms and sleep habits with the use of previous validated questionnaires, the Aberrant Behavior Checklist (ABC) [14] and the Children’s Sleep Habits Questionnaires (CSHQ) [15], respectively. Information regarding cognitive activity and adaptive skills were obtained from previous school evaluation records, documenting cognitive activity (by standard measures such as Woodcock-Johnson III test) and adaptive skills (by standard measures such as Vineland Adaptive Behavior Scale (VABS) [16]. These were data documented within 1 year of enrollment to the study. In some ASD patients, adaptive skills were also assessed by VABS in the clinic.

#### Non-ASD controls

Typically developing, non-ASD control subjects were ecruited in the Pediatric Allergy/Immunology Clinic.

Demographic information of the study subjects is summarized in Table 1.

**Table 1:** Demographic information of the study subjects

Study group	Age (year)	Gender (male/female)	Ethnicity
	Median (range)		
ASD subjects (*N* = 69)	11.8 (2.8–27.0)	52:16	1 AA, 6 Asians, 2 mixed, 90 W
Normal control (*N* = 27)	10.1 (3.6–27.0)	16:11	3 Asians, 2 mixed, 22 W

Abbreviations: *AA* African American, *ASD* autism spectrum disorder, *W* Caucasian

#### Diagnosis of food allergy (FA)

IgE-mediated FA was diagnosed with reactions to offending food, by affecting skin, GI, and/or respiratory tract immediately after intake of offending food (within 2 h), supported by prick skin testing (PST) reactivity, and/or the presence of food allergen-specific IgE in the serum. Non-IgE-mediated food allergy (NFA) was diagnosed with resolution of GI symptoms following implementation of a restricted diet (i.e., avoidance of offending food), and recurrence of symptoms upon re-introduction of offending food, following the Food Allergy Diagnostic Guidelines [17]. NFA patients are per definition, nonreactive to PST, and negative for food allergen-specific, serum IgE [17].

#### Diagnosis of asthma and allergic rhinitis

Allergic rhinitis (AR) and allergic conjunctivitis (AC) were diagnosed with positive PST reactivity, and/or the presence of allergen-specific IgE in the serum, accompanied by clinical features consistent with AR and AC [18, 19]. Asthma diagnosis was based on the guidelines from the Expert Panel Report 3 [20]. Asthma, without PST reactivity to allergens and/or allergen-specific IgE antibodies, was categorized as non-atopic asthma [19].

#### Antibody deficiency syndrome

Specific polysaccharide antibody deficiency (SPAD) was diagnosed by the absence of detectable antibody (Ab) titers (more than 1.3 μg/mL) to more than 11 of 14 serotypes of *Streptococcus pneumonia*, following a booster dose of Pneumovax® [21], a standard diagnostic measure for SPAD.

### Sample obtainment

Peripheral blood (PB) samples were obtained by venipuncture after the obtainment of informed consent. Efforts were made to obtain the PB samples at the time of routine blood work in order to minimize the numbers of venipuncture in all the study subjects. For the non-ASD control subjects, only 1 sample was obtained. For ASD subjects with fluctuating behavioral symptoms and varying GI symptoms, we attempted to obtain at least 2 samples, one when behavioral symptoms were at what was considered their baseline and another when parents reported exacerbation of behavioral symptoms. Venipuncture was conducted by the physician, and if requested, the site of venipuncture was numbed by applying a topical lidocaine/prilocaine cream (EMLA® cream).

### Cell cultures

Peripheral blood mononuclear cells (PBMCs) were isolated by Ficoll-Hypaque density gradient centrifugation. PBMo were purified by negatively selecting PBMo depleting T, B, natural killer, and dendritic cells from PBMCs, using magnetic beads labeled with anti-CD3, CD7, CD16, CD19, CD56, CD123, and glycophorin A (monocyte separation kit II human, MILTENYI BIOTEC, Cambridge, MA).

Cytokine production by PBMo was assessed by incubating purified PBMo (2.5 × 10^5^ cells/ml) overnight with a Toll-like receptor (TLR)4 agonist (LPS; 0.1 μg/ml, GIBCO-BRL, Gaithersburg, MD), a TLR2/6 agonist (zymosan; 50 μg/ml, Sigma-Aldrich, St. Luis, Mo), a TLR7/8 agonist (CL097, water-soluble derivative of imidazoquinoline, 20 μM, InvivoGen, San Diego, CA), and a dectin 1 agonist [heat killed *Candida albicans* as a source of ß-lactam (10^9^ cells/ml)—10 μl/ml, InvivoGen] in RPMI 1640 with additives as previously described [22]. Overnight incubation was adequate to induce the optimal responses in this setting.

Levels of pro-inflammatory [tumor necrosis factor-α (TNF-α), interleukin-1ß (IL-1β), IL-6, IL-12p40, and IL-23] and counter-regulatory [IL-10, transforming growth factor-ß (TGF-ß), and soluble TNF receptor II (sTNFRII)] cytokines in the culture supernatant were measured by enzyme-linked immuno-sorbent assay (ELISA). The ELISA OptEIA™ Reagent Sets for IFN-γ, IL-1ß, IL-5, IL-6, IL-10, IL-12p40, and TNF-α (BD Biosciences) and for sTNFRII, IL-17 (IL-17A), and TGF-ß were obtained from BD Biosciences and R&D (Minneapolis, MN), respectively. IL-23 ELISA kit was purchased from eBiosciences, San Diego, CA. Intra- and inter-variations of cytokine levels were less than 5%.

### Sequencing of miRNA

miRNAs were extracted using the miRNAeasy kit (Quiagen, Valencia, CA). The bar-coded small RNA libraries were prepared with Ion Total RNA-Seq Kit V2 (Life Technologies, Grand Island, NY) and Ion Xpress™RNA-Seq Barcode 1-16 Kit (Life Technologies). For template preparation, generated libraries were clonally amplified and beaded using the Ion One Touch 2 system (Life Technologies). The resulting templates were sequenced using Ion 318 chips (life Technologies). Sequence reads were processed in the Torrent Server v4.4 (Life Technologies) and exported to the STRAND NGS 2.7 (Strand Genomics, Inc., San Francisco, CA) for data analysis. After purification of miRNA in the PI’s laboratory, miRNA profiling was conducted under the supervision of Drs. J. Dermody and Gocke Toruner, co-authors, at the Institute of Genomic Medicine, Rutgers-NJMS. The difference of miRNA expression between groups as fold differences, and heat map analysis with the use of *Z*-scores was performed with the use of the software Strand NGS® (Strandngs, San Francisco, CA).

### miRNA target gene analysis

For determining the targeted genes by specific miRNAs, microRNA Data Integration portal (mirDIP) was used (http://ophid.utoronto.ca/mirDIP/index.jsp) [23].The putative gene targets with integrated score of 0.3 and higher were further analyzed using Database for Annotation, Visualization and Integrated Discovery (DAVID) (https://david.ncifcrf.gov/home.jsp) [23]. Functional annotation analysis was performed to see enrichment for genes belonging to specific KEGG [24] pathways and UniProt and gene ontology keywords. Only categories scored *p* < 0.05 after Benjamini-Hochberg multiple hypothesis correction were reported as significant in the “Results” sections. All data associated with analysis can be found in the Additional files.

### Statistical analysis

For comparison of cytokine/ABC score values within the several groups, the one-way analysis of variance (ANOVA) was used if the data distributed normally. If the data are not normally distributed, difference in multiple groups was analyzed by Kruskal-Wallis test. For differences in frequency between the two groups, Fisher’s exact test was used. For correlation of two data sets, Spearman’s test was used. A *p* value of < 0.05 was considered nominally significant.

## Results

### Subgrouping ASD samples on the basis of IL-1ß/IL-10 ratios produced differences of miRNA expression in ASD monocytes 

**Fig. 1:**
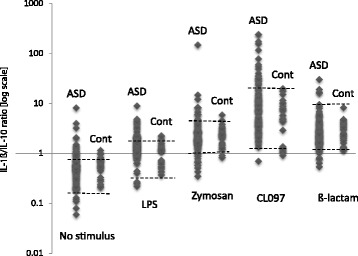
IL-1β/IL-10 ratios under cultures without a stimulus and with stimuli (LPS, zymosan, CL097, and β-lactam). − 1 SD and + 1 SD values of reference values previously obtained from 33 normal controls are shown as dotted lines

Previously, we have observed marked variation in the production of IL-1ß and IL-10 by PBMo in a subset of ASD subjects [9]. These changes are more easily appreciated when expressed as IL-1ß/IL-10 ratios, in parallel with changes in behavioral symptoms [25]. In this study, we examined IL-1ß/IL-10 ratios produced by the ASD PBMo in comparison with non-ASD PBMo, as shown in Fig. 1. Fluctuating IL-1ß/IL-10 ratios in ASD PBMo were observed in some ASD subjects, a finding that is consistent with our previous study [25]. It should be noted that in selected ASD subjects (*N* = 23), PBMo samples were obtained 2–3 times. Each sample was analyzed for both cytokine production and miRNA expression, along with an evaluation of behavioral symptoms using the ABC checklist. This is due to the fact that we have previously observed changes in cytokine production by ASD PBMo in association with changes in behavioral symptoms [9]. Non-ASD control cells revealed the similar tight ranges of IL-1ß/IL-10 ratios, as we have reported before [25]. Thus, ASD monocyte samples examined in this study revealed a much higher frequency of high/low IL-1ß/IL-10 ratios that were either higher or lower than non-ASD control cells (*p* < 0.0005 by Fisher’s exact test). Therefore, we subdivided the ASD PBMo samples into high, normal, and low IL-1ß/IL-10 ratio groups, defining as described below [25]:

#### High IL-1ß/IL-10 ratio

IL-1ß/IL-10 ratios > +2SD than control cells under at least 1 culture condition and/or > +1SD under more than 2 culture conditions.

#### Normal IL-1β/IL-10 ratio

IL-1ß/IL-10 ratios fall into − 1 SD < IL-1ß/IL-10 ratios < +1SD under all the culture conditions or + 1 SD < IL-1ß/IL-10 ratios < +2SD under only 1 culture condition.

#### Low IL-1β/IL-10 ratios

IL-1ß/IL-10 ratios < − 1 SD under at least 1 culture condition.

### Differences of miRNA expression in ASD monocytes on the basis of IL-1ß/IL-10 ratios

Differences in miRNA expression in each ASD subgroup as described above was examined in comparison with miRNA expression by non-ASD control cells, as well as between the above-described ASD groups.

ASD cells submitted for miRNA sequencing included cells with high ratio group (*N* = 43), low ratios (*N* = 18), and normal ratios (*N* = 47).

ASD cells with high IL-1ß/IL-10 ratios revealed upregulated expression of multiple miRNAs, as compared to other ASD groups and non-ASD control cells (Table 2). In contrast, ASD cells with normal ratios revealed little differences from non-ASD controls (Table 2). ASD cells with low ratios revealed upregulation of 1 miRNA and downregulation of 2 miRNAs (Table 2). When all ASD samples were combined, no change in miRNA expression was observed, as compared to non-ASD controls. All the miRNAs revealed over 2-fold changes between the designated 2 groups are shown in Additional file 1. Table 3 summarizes miRNAs expressed at least more than 3-fold higher or lower in ASD cells with high/low IL-1ß/IL-10 ratios, as compared to non-ASD controls.

**Table 2:** Differences of microRNA (miRNA) expression in ASD subjects with high, normal, and low IL-1ß/IL-10 ratio

	miRNA numbers	miRNA numbers
	Upregulated	Downregulated
Comparison between ASD samples		
High ratio^a,b^ vs. low ratio	21	0
	> 4-fold higher 3^a^	
	> 3-fold higher 3	
High ratio vs. normal ratio	19	0
	> 4-fold higher 4	
	> 3-fold higher 2	
Low ratio vs. normal ratio	0	0
Comparison with normal controls		
High ratio vs. controls	7	0
	> 3-fold higher 2	
Low ratios vs. controls	1	2
		> 3-fold lower 1
Normal ratios vs. controls	0	1
All ASD samples vs. controls	0	0

^a^High, normal, and low IL-1ß/IL-10 ratio groups were defined in the 1st paragraph of the “Results” section

^b^Numbers of ASD samples submitted for miRNA sequencing are the following: high ratio group (*N* = 43), low ratio group (*N* = 18), and normal ratio group (*N* = 47). Among non-ASD control cells (*N* = 27), 23 samples revealed normal IL-1ß/IL-10 ratios, 2 samples revealed high ratios with > + 1 SD in 2 culture conditions, and 2 samples had low ratios with < − 1 SD in 2 culture conditions. Thus, ASD samples examined in this study revealed much higher frequency of high/low IL-1ß/IL-10 ratios, as compared to non-ASD control cells (*p* < 0.0005 by Fisher’s exact test)

**Table 3:** miRNAs with notable changes between groups

miRNAs	Changes observed
miR-342	Higher in ASD cells with high IL-1ß/IL-10 ratios as compared all other study groups Lower in ASD cells with low and normal IL-1ß/IL-10 ratios as compared to controls
miR-181a-1/2	Higher in ASD cells with high IL-1ß/IL-10 ratios as compared all other study groups
miR-93	Higher in ASD cells with high IL-1ß/IL-10 ratios as compared all other study groups
miR-223	Higher in ASD cells with high IL-1ß/IL-10 ratios as compared all other study groups
miR-1248	Higher in ASD cells with high or low IL-1ß/IL-10 ratios as compared to controls

**Fig. 2:**
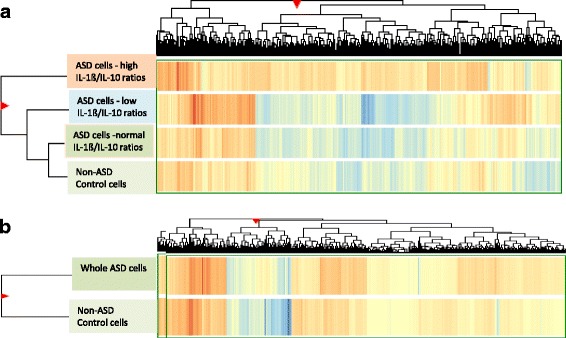
Heat map analysis results of miRNA expression by ASD monocytes with high, normal, and low IL-1ß/IL-10 ratios, in comparison with non-ASD control monocytes (panel A) and heat map analysis results of whole ASD samples vs. non-ASD controls (panel B). Significant difference in miRNA expression between ASD vs. non-ASD control cells only became apparent, when the cells were subdivided, on the basis of IL-1ß/IL-10 ratios

Difference of miRNA expression in these 3 ASD subgroups in comparison with non-ASD controls was analyzed with the use of *Z*-score heat map (Fig. 2). Our results supported our initial results; ASD cells with high IL-1ß/IL-10 ratios revealed the most significant differences in miRNA expression, as compared to non-ASD controls, while ASD cells with normal ratios revealed the least difference (see Additional files 2 and 3 for *Z* test results in detail).

### miRNA target gene analysis results

miRNA target gene analysis was done between groups when initial mirDIP analysis identified target genes. The detailed results of *Z* test are shown in Additional files 4, 5, and 6 (see Additional files 4, 5, and 6 for groups A, B, and C analysis, respectively).

#### ASD cells with high IL-1ß/IL-10 ratios vs. ASD cells with normal ratios (group A)

One thousand four hundred sixty-one putative targets were identified by mirDIP. Further analysis with DAVID software revealed that genes on Ras signaling (*p* = 1 × 10^−3^), MAPK signaling (*p* = 1 × 10^−3^), and PI3K-AKT signaling (p = 1 × 10^−2^) pathways were overrepresented among the submitted gene targets. In addition, functional analysis clustering indicated genes with UniProt keywords such as transcription regulation (*p* = 7.11 × 10^−11^), transcription (*p* = 7.68 × 10^−11^), zinc finger (*p* = 1.4 × 10^−6^), cell junction (*p* = 5.9 × 10^−6^), synapse (*p* = 3.8 × 10^−4^), post-synaptic membrane (*p* = 7 × 10^−7^), and gene ontology terms like cell junctions (*p* = 3.×10^−4^), cell-cell adhesion (*p* = 7.2 × 10^−4^), and cell adherens junctions (*p* = 1.4 × 10^−4^) are overrepresented.

#### ASD cells with higher IL-1ß/IL-10 ratio vs. ASD cells with low ratios (group B)

One thousand two hundred thirty-four putative targets were identified by mirDIP. Further analysis with DAVID software revealed that genes on Ras signaling (*p* = 4.5 × 10^−5^) and MAPK signaling (*p* = 2.7 × 10^−2^) pathways were overrepresented among the submitted gene targets. In addition, functional analysis clustering indicated genes with UniProt keywords such as transcription regulation (*p* = 4.1 × 10^−8^), transcription (*p* = 4.9 × 10^−8^), zinc finger (*p* = 2.9 × 10^−6^), cell junction (*p* = 1.54 × 10^−8^), synapse (*p* = 2.8 × 10^−5^), autism spectrum disorder (*p* = 1.9 × 10^−4^), and gene ontology terms like cell junctions (*p* = 2.5 × 10^−4^), cell-cell adhesion (*p* = 2.5 × 10^−4^), and cell adherens junctions (*p* = 7.9 × 10^−3^) are overrepresented.

#### ASD cells with high IL-1ß/IL-10 ratios vs. non-ASD control cells (group C)

Two hundred sixty-three putative targets were identified by mirDIP. Further analysis with DAVID software revealed no enrichment of a specific KEGG pathway. Functional analysis clustering indicated genes with UniProt keywords such as transcription regulation (*p* = 2.8 × 10^−5^), transcription (*p* = 2.7 × 10^−5^), cell junction (*p* = 1 × 10^−2^), and synapse (*p =* 1 × 10^−2^) are overrepresented.

These results are consistent with potential impact of differential miRNA changes through genes important in key signal transduction pathways, zinc-finger domain transcription, and molecules important in formation of synaptic junctions.

### Changes in inflammatory and counter-regulatory cytokine expression in ASD cells on the basis of IL-1ß/IL-10 ratios

Since the miRNAs that were upregulated in the ASD cells with high IL-1ß/IL-10 ratios were those associated with regulation of key signaling pathways of inflammation, we examined the changes of inflammatory and counter-regulatory cytokines produced by PBMo under all culture conditions tested. The results are summarized in Table 4.

**Table 4:** Cytokine production by purified ASD PBMo with high, normal, or low IL-1ß/IL-10 ratios

Cytokine measured	Stimulant	ASD—high (*N* = 43)	IL-1β/IL-10 ratio ASD—normal (*N* = 47)	ASD—low (*N* = 18)	Non-ASD cells (*N* = 27)
IL-1ß^a^	Medium	628.7 ± 455.9	420.8 ± 210.2	206.0 ± 191.2	306.2 ± 199.3
	LPS	2518.0 ± 1012.0	1968.0 ± 460.2	1407.2 ± 586.7	1968.2 ± 460.2
	Zymosan	2640.2 ± 958.0	2205.6 ± 715.2	1682.6 ± 951.3	1872.0 ± 692.1
	CL097	3470.2 ± 917.0	3008.8 ± 848.0	2836.2 ± 1126.3	2257.1 ± 1040.7
	ß-lactam	2452.2 ± 1056.3	2108.3 ± 713.6	1725.3 ± 924.6	1726.2 ± 566.9
IL-6^b^	Medium	3701.5 ± 1746.2	3784.4 ± 1137.1^8^	2829.1 ± 1197^j^	2892.2 ± 1258.0
	LPS	23,478.1 ± 9649.6	22,071.3 ± 10,171.0	24,177.8 ± 10,285.6	18,459.5 ± 10,120.8
	Zymosan	4945.1 ± 1406.1	5064.0 ± 841.9	5147.9 ± 1705.8	5050.3 ± 1068.3
	CL097	4025.3 ± 1335.0	4382.5 ± 988.2	5180.6 ± 2170.4	4111.3 ± 1556.5
	ß-lactam	4842.3 ± 1238.1	4780.5 ± 1048.8	5130.9 ± 1935.2	4641.6 ± 1184.9
TNF-α^c^	Medium	95.7 ± 100.2	101.3 ± 139.3	49.0 ± 70.0^i^	73.7 ± 114.3
	LPS	478.9 ± 373.9	509.5 ± 411.5	196.8 ± 194.9^i^	363.8 ± 399.7
	Zymosan	716.4 ± 463.4	731.0 ± 546.0	309.0 ± 241.8^6^	774.4 ± 587.8
	CL097	1148.1 ± 900.3	1304.7 ± 843.2	889.4 ± 534.9^e^	859.6 ± 685.2
	ß-lactam	1200.5 ± 859.9	1136.5 ± 681.8	689.2 ± 440.3^6^	1061.2 ± 565.5
IL-10^a^	Medium	678.5 ± 411.1	789.6 ± 319.9^8^	741.8 ± 447.7	533.4 ± 301.8
	LPS	1195.0 ± 338.7	1444.9 ± 325.2^8^	1556.6 ± 465.5^8^	1239.8 ± 358.0
	Zymosan	851.9 ± 388.4	1144.6 ± 339.1^8^	1166.0 ± 545.0^8^	874.5 ± 381.1
	CL097	235.3 ± 197.5	683.4 ± 408.5^8^	924.8 ± 549.0^8^	491.4 ± 294.7
	ß-lactam	674.1 ± 345.9	994.7 ± 355.5^8^	1126.5 ± 549.0^8^	740.7 ± 412.0
TGF-ß^d^	Medium	300.8 ± 232.5	452.5 ± 341.8	418.2 ± 232.6	421.1 ± 263.1
	LPS	301.3 ± 232.3	424.0 ± 332.3	407.2 ± 253.9	432.4 ± 266.6
	Zymosan	257.8 ± 212.2	360.1 ± 302.0	366.1 ± 216.7	375.5 ± 225.5
	CL097	192.0 ± 127.7	363.3 ± 316.4	370.1 ± 300.1	397.5 ± 280.7
	ß-lactam	245.0 ± 199.6	314.3 ± 201.4	403.7 ± 308.6	331.6 ± 182.7

^a^As expected, IL-1ß and IL-10 levels differ among the ASD cell groups with high, normal, and low IL-1ß/IL-10 ratios and non-ASD control cells (*p* < 0.005 by one-way ANOVA with the use of log-transformed data)

^b^IL-6 level production without stimuli differed among the groups tested, reflecting a higher IL-6 production by ASD cells with high and normal IL-1ß/IL-10 ratios (*p* < 0.05 by one-way ANOVA with the use of log-transformed data)

^c^NF-α levels differed among groups under LPS- and CL097-stimulated cultures by one-way ANOVA with the use of log-transformed data (*p* < 0.01 for LPS and *p* < 0.05 for CL097)

^d^TGF-ß levels differed among groups under cultures without stimuli (*p* < 0.05) and under CL097-stimulated cultures (*p* < 0.005) by one-way ANOVA with the use of log-transformed data

Since our initial subgrouping of ASD PBMo was based on IL-1ß/IL-10 ratios, the observed increase in IL-1ß production and decrease in IL-10 production in ASD PBMo with high IL-1ß/IL-10 ratios were expected. However, we also observed an increase in the production of TNF-α and IL-6, an inflammatory cytokine, and a decrease in the production of TGF-ß, a counter-regulatory cytokine in ASD PBMo with high ratios. In ASD PBMo with low IL-1ß/IL-10 ratios, we observed a decrease in the production of both IL-1ß and TNF-α and an increase in IL-10 (Table 4). In addition, ASD PBMo with normal ratios still produced higher levels of IL-1ß and IL-10 than non-ASD cells and ASD cells with low ratios (Table 4). These results indicate that there are changes in production of other cytokines, other than IL-1ß and IL-10.

### Changes in behavioral scores in ASD subjects when ASD cells reveals high, normal, or low IL-1ß/IL-10 ratios

Previously, we observed an association between changes in cytokine levels produced and certain behavioral symptoms in some ASD subjects who had fluctuating behavioral symptoms [9]. This study also addressed whether behavioral symptoms, assessed by ABC subscale scores, differ on the basis of changes in IL-1ß/IL-10 ratios; ABC questionnaires were filled out at the time of each PBMo obtainment, prior to venipuncture; it takes only 5–10 min to filling out the ABC questionnaires. For a selected set of ASD subjects, we obtained multiple PBMo samples, and ABC questionnaires were completed prior to each PBMo sampling. A summary of ABC subscale scores is shown in Table 5. Lower scores of subscale II (lethargy) were observed in ASD subjects when their PBMo revealed low IL-1ß/IL-10 ratios (Table 5), while subscale V (inappropriate speech) scores were lower in ASD subjects when their PBMo had high ratios (Table 5).

**Table 5:** ABC subscale scores between ASD groups with high, normal, or low IL-1ß/IL-10 ratios^a^

	IL-1ß/IL-10 ratio			Kruskal-Wallis test
ABC subscale	High	Normal	low	*p* value
I	15.8 ± 6.9^a^	14.7 ± 10.0	9.3 ± 5.6	0.1007
II	11.2 ± 7.2	11.3 ± 9.7	5.5 ± 5.3	0.0493^b^
III	8.2 ± 4.9	6.7 ± 5.1	6.5 ± 5.4	0.3996
IV	18.9 ± 10.2	18.3 ± 10.5	14.7 ± 6.9	0.4485
V	2.9 ± 2.8	5.1 ± 3.5	5.0 ± 4.2	0.0443^b^

^a^Sample numbers are shown in Table 4

^b^Difference noted among groups, reflecting low ABC subscale II (Hyperactivity) in the low IL-1ß/IL-10 ratio groups and low ABC subscale V (inappropriate speech) in the high IL-1ß/IL-10 ratio groups

### Association between ABC scores and cytokine levels produced by ASD PBMo

We then examined whether there was an association between ABC scores and changes in levels of inflammatory vs. counter-regulatory cytokines when ASD PBMo samples were divided into groups with high, normal, and low IL-1ß/IL-10 ratios. We reasoned that if innate immune responses play a role in ASD behavioral symptoms, we would be able to detect associations between ABC scores and cytokine levels produced by ASD PBMo, major innate immune cells in the periphery. We also reasoned that such an association may only be detected when innate immune responses deviated from normal responses. Therefore, high or low IL-1ß/IL-10 ratios were used as a marker for deviated innate immune responses. Our results revealed negative associations of between both IL-6 and IL-1ß levels under cultures with stimuli and ABC subscale I (irritability) scores in the low IL-1β/IL-10 ratio group (Table 6). In this group, without a stimulus, irritability scores were positively associated with the levels of IL-1ß and TNF-α (Table 6). In both the normal and high IL-1ß/IL-10 groups, such an association was less evident (Table 6). A similar tendency was also observed between cytokine levels and ABC subscale II (Lethargy) and IV (hyperactivity) scores, although results were less significant (data not shown). In the high IL-1βIL-10 ratio group, ABC subscale III (stereotypy) scores were positively associated with levels of two inflammatory cytokines, IL-1ß and IL-6, under cultures stimulated with zymosan and CL097. However, such an association was not observed in either the normal or low IL-1ß/IL-10 ratio groups (Table 7). These results indicate that the effects of cytokines on ASD behavioral symptoms may be altered in parallel with changes in IL-1ß/IL-10 ratios.

**Table 6:** Correlation between cytokine levels and ABC subscale 1 (irritability) score

Irritability (ABC I)		IL-1ß/IL-10 ratio		
Cytokines measured^a^	Simulant	High (*N* = 43)	Normal (*N* = 43)	Low (*N* = 18)
IL-1ß	None	− 0.0693^a^	− 0.0966	0.5841 (*p* < 0.05)
	LPS	− 0.1675	− 0.2769	− 0.323
	Zymosan	− 0.2907	− 0.0977	− 0.6969 (*p* < 0.01)
	CL097	− 0.0535	− 0.1543	− 0.4646
	ß-lactam	− 0.4134 (*p* < 0.02)	− 0.2494	− 0.5575 (*p* < 0.05)
IL-6	None	− 0.1135	0.0348	− 0.0089
	LPS	0.0029	− 0.1326	− 0.6018 (*p* < 0.05)
	Zymosan	− 0.0155	0.1598	− 0.6859 (*p* < 0.01)
	CL097	− 0.1148	− 0.0457	− 0.7633 (*p* < 0.005)
	ß-lactam	− 0.0524	0.0015	− 0.7307 (*p* < 0.005)
TNF-α	None	0.0672	0.2512	0.8359 (*p* < 0.005)
	LPS	− 0.061	0.3533 (*p* < 0.05)	0.6903 (*p* < 0.01)
	Zymosan	− 0.0133	0.3491 (*p* < 0.05)	− 0.0376
	CL097	0.0311	0.1123	0.2257
	ß-lactam	− 0.0789	0.2143	0.1305
IL-10	None	− 0.0072	0.0938	0.4956
	LPS	− 0.2519	− 0.2478	0.2102
	Zymosan	− 0.1111	− 0.1858	0.1925
	CL097	− 0.0733	− 0.1351	− 0.3031
	ß-lactam	− 0.2178	− 0.3277	0.323

^a^Correlation coefficient by Spearman’s test between cytokine levels and scores of ABC subscale I (irritability). Stimulants used for cultures are shown in the column 2

**Table 7:** Correlation between cytokine levels and ABC subscale 3 (stereotypy) score

Stereotypy (ABC III)		IL-1ß/IL-10 ratio		
Cytokines measured	Stimulants	High (*N* = 43)	Normal (*N* = 47)	Low (*N* = 18)
IL-1ß	None	0.0104^a^	− 0.1728	0.1151
	LPS	0.1351	− 0.0961	− 0.1328
	Zymosan	0.3671 (*p* < 0.05)	− 0.0671	0.146
	CL097	0.5686 (*p* < 0.005)	− 0.0675	0.1416
	ß-lactam	0.327	− 0.1262	− 0.3518
IL-6	None	0.0058	− 0.0023	− 0.0664
	LPS	0.3605 (*p* < 0.05)	− 0.294	− 0.2102
	Zymosan	0.3321	0.1932	− 0.2323
	CL097	0.4092 (*p* < 0.02)	− 0.2431	0.0376
	ß-lactam	0.4533 (*p* < 0.01)	0.0016	− 0.0066
TNF-α	None	0.0037	0.065	− 0.0944
	LPS	0.2059	0.2085	− 0.0243
	Zymosan	0.2343	0.2385	0.1416
	CL097	0.2302	0.0841	− 0.312
	ß-lactam	0.1778	0.1369	− 0.3429
IL-10	None	0.0849	0.0562	0.4867
	LPS	− 0.1605	0.0094	0.5155 (*p* = 0.0592)
	Zymosan	− 0.0049	0.2030	0.1018
	CL097	0.2465	− 0.1753	− 0.0288
	ß-lactam	− 0.0879	0.1662	0.0133

^a^Correlation coefficient by Spearman’s test between cytokine levels and scores of ABC subscale III (stereotypy). Stimulants used for cultures are shown in the column 2

### Differences in clinical features in ASD subjects on the basis of IL-1ß/IL-10 ratios

We have reported before that certain ASD subjects revealed high or low IL-1ß/IL-10 ratios, depending on the time points when the sample was obtained, while others’ IL-1ß/IL-10 ratios remained stable [25]. The ASD subjects whose IL-1ß/IL-10 ratios remained stable generally fell into the group with normal IL-1ß/IL-10 ratios in our previous observation. Therefore, we also assessed differences in clinical features of ASD subjects enrolled in this study, on the basis of high/low IL-1ß/IL-10 ratios vs. normal ratios. Clinical features assessed include cognitive and adaptive skills and frequency of childhood illnesses associated with immune-mediated inflammation. The results revealed that ASD subjects with high/low IL-1ß/IL-10 ratios had a higher frequency of NFA and lower adaptive skills (Table 8). They also tended to reveal higher frequencies of seizure disorders and specific antibody deficiency, although this was not statistically significant, most likely due to low subject numbers. When we assessed ASD clinical features solely based on IL-1ß/IL-10 ratios in a larger numbers of subjects, we observed significantly higher frequencies of seizure disorders and antibody deficiency syndrome in ASD subjects with high/low IL-1ß/IL-10 ratios [25]. It should be noted that in non-ASD control subjects, no asthma, seizure disorders, or specific antibody deficiency were reported. Two non-ASD control subjects had history of NFA which was fully resolved at the time of sample obtainment.

**Table 8:** Summary of clinical features of ASD subjects with high/low IL-1ß/IL-10 ratios vs. ASD subjects with normal ratios

Clinical characteristics	ASD subjects IL-1ß/IL-10 ratio High or low (*N* = 43)	ASD subjects IL-1ß/IL-10 ratio Normal (*N* = 26)	Fisher’s exact test
Age median, year (range)^a^	12.1 (3.3–27.0)	11.9 (3.8–22)^a^	
Gender (male/female)	33:10	20:6	1.0
Cognitive skills < 1st %	33/43 (76.7%)	17/26 (65.3%)	0.4055
Social skills^b^ < 1st %	40/43 (93.0%)	18/26 (69.2%)	0.0154
NFA^c^	31/43 (72.1%)	11/26 (42.3%)	0.0373
Seizures	7/43 (16.3%)	1/26 (3.8%)	0.243
Asthma^d^	5/43 (11.6%)	1/26 (3.8%)	0.3978
Allergic rhinitis	7/43 (16.3%)	2/26 (7.7%)	0.4664
Antibody deficiency	8/43 (18.6%)	2/26 (7.7%)	0.2993

^a^Age entered to the study

^b^Social skills were based on school assessment and VABS scores conducted at school or at our clinic

^c^Non-IgE-mediated food allergy (NFA) and diagnosis of NFA are detailed in the methods section

^d^Non-ASD controls employed in this study were all developing typically without history of asthma, seizure disorder, or antibody deficiency. Allergic rhinitis is reported in 2 non-ASD controls. Two non-ASD subjects reported a history of NFA, but they were tolerating a regular diet at the time of sample obtainment, following complete resolution of NFA symptoms

## Discussion

Our study revealed that changes in miRNA expression by ASD PBMo paralleled changes in IL-1β/IL-10 ratios (higher or lower than non-ASD control cells). The miRNAs altered in expression are those that affect key signaling pathways mediating or regulating inflammation processes. In addition, changes in IL-1β/IL-10 ratios were also associated with production of inflammatory and counter-regulatory cytokines other than IL-1β or IL-10 (Table 4). Associations between ASD behavioral symptoms (assessed by ABC) and cytokine levels were also found to change, in parallel with changes in IL-1ß/IL-10 ratios (Tables 6 and 7). Our results indicate that IL-1ß/IL-10 ratios from ASD PBMo could serve as biomarkers for immune-mediated inflammation in some ASD subjects, in association with changes in miRNA expression.

High-throughput RNA sequencing revealed the existence of a large number of noncoding RNAs in the human genome and regulatory roles of evolutionally conserved short noncoding RNA or microRNA (miRNA) [26]. miRNAs control post-transcriptional gene expression by repressing translation or promoting degradation of messenger RNA (mRNA) by biding to the 3′UTD of target mRNAs [26]. Monocyte and macrophage lineage cells play crucial roles in tissue inflammation and subsequent injury repair in multiple organs, including the brain [27, 28]. Interestingly, differentiation and cellular functions of monocyte/macrophage lineage cells are tightly regulated by miRNAs [29].

As discussed briefly in the introduction, immune-mediated inflammation has been implicated in the onset and progress of ASD. However, the fact that immune abnormalities reported in ASD children affect almost every arm of the immune system makes it difficult to understand the role that the immune system plays [7, 30]. On the other hand, findings of such varied immune abnormalities in ASD suggest that there may be impairments in the key signaling pathways that broadly affect the immune system. In that regard, the recently described animal model of autism with the use of germline mislocalization of PTEN [10] is intriguing. PTEN expression is widely regulated by the phosphatase network and is affected by upstream cytokines such as IL-1ß, produced by innate immune cells. PTEN expression is also known to be regulated by multiple miRNAs that can also be up- or downregulated by inflammatory mediators [31–34]. Resultant PTEN-mediated changes in the phosphatase network can affect differentiation of T lineage cells, especially Treg cells as well as mitochondrial functions. It should be noted that patients suffering from congenital PTEN mutation (PTEN hamartoma tumor syndrome) suffer from various abnormalities of T and B lineage cells, autoimmune conditions, and mitochondrial dysfunctions [11]. Broad changes in the immune system may also affect the brain’s functions as well. In fact, we previously reported an association between ASD behavioral symptoms and monocyte cytokine production profiles in a subset of ASD children [9]. Others also reported that both innate immune responses and T cell activation status are associated with more severe developmental impairment and/or aggressive behaviors in ASD subjects [35].

Given these findings, we initially hypothesized that changes in cytokine production profiles by PBMo could affect other lineage cells through mediators released by PBMo. However, most cytokines released by PBMo have short half-lives (less than 1–2 h) [36]. Thus, effects from cytokines released by PBMo may be limited to cells in the vicinity or via inflammatory refluxes through afferent nerves to the brain [37]. Alternatively, activated PBMo may migrate to target organs, such as the brain, and change into tissue macrophages [38]. However, this may take time and may not explain initial, swift effects of the innate immune responses on multiple organs. Then, are there any other means by which macrophage/monocyte lineage cells can utilize for intercellular communications?

Recently, miRNAs emerged as major mediators of intercellular communications exerted by monocyte/macrophage lineage cells [39]. Monocyte/macrophage lineage cells secrete extracellular membrane vesicles that contain proteins, mRNA, and miRNAs; they are called microvesicles (MVs). Next to platelets, monocytes/macrophage lineage cells are the 2nd most common contributors for MVs in the peripheral circulation [40]. miRNAs are stable in MVs and serve as mediators of intercellular communications to cells that are not closely located to these innate immune cells [29, 39]. In this way, local innate immune responses may exert action on multiple organs that are remotely located. The initial step to test this possibility is to determine whether miRNA expression changes in parallel with changes in IL-1β/IL-10 ratios. Thus, we examined miRNA expression in high, normal, or low IL-1ß/IL-10 ratio groups in ASD PBMo.

Previously, we found that the most notable differences in cytokine profiles by ASD vs. non-ASD control PBMo were in the production of IL-1β, an inflammatory cytokine, and IL-10, a counter-regulatory cytokine. In non-ASD control cells, we observed less variable levels of IL-1ß and IL-10, along with a tight positive association between IL-1ß and IL-10 [9, 25] and as was the case for control cells employed in this study (Fig. 1). In contrast, we found highly variable production of IL-10 and IL-1ß by ASD PBMo, resulting in variable IL-1ß/IL-10 ratios (Fig. 1). In addition, the IL-1ß/IL-10 ratios can vary in the same ASD subject, and the ratios also appear to have an association with behavioral changes in ASD subjects, depending on the time of sampling [25].

Given the regulatory role of miRNA in monocyte functions, as well as their role in intercellular communications, we hypothesized that PBMo from ASD subjects with high/low IL-1ß/IL-10 ratios reveal changes in expression of miRNAs that affect major signaling pathways controlling inflammatory processes. Indeed, we found up- or downregulation of miRNAs in ASD PBMo with high/low IL-1ß/IL-10 ratios. Interestingly, miR-181a is reported to regulate inflammatory responses in monocyte/macrophage lineage cells, partly through downregulating inflammatory cytokines [41] and suppressing downstream signaling pathways involving PTEN [33, 42]. MiR-181a is also reported to affect mitochondrial functions and tolerance induction by T cells [43, 44]. On the other hand, miR-93 reportedly activates the PI3K/Akt pathway, inhibiting translation of upstream genes including PTEN and PHLPP2 [32, 34]. This may result in a similar immune-dysregulated status that is found in patients with PTEN mutation [11]. MiR-342 has been implicated with murine macrophage survival [45] and affects tissue repair and cell differentiation via TGF-ß signaling through Notch pathway [46, 47]. miR-223 is reportedly associated with MV secretion by monocyte/macrophage lineage cells [40] and implicated in the pathogenesis of inflammatory bowel disease [48]. Reports on the functions of miR-1248 are limited, but one report indicated that miR-1248 over-expression in HeLa cells, a cervical cancer cell line, resulted in upregulation of inflammatory cytokines (IL-6 and IL-8) and other markers of inflammation [49]. It should be noted that miRNA target gene analysis did support changes in miRNA expression affecting key signal transduction pathways, zinc-finger domain transcription, and molecules important in formation of synaptic junctions. Although our data were obtained from monocytes, our results may indicate changes in miRNA expression affecting both the immune system and the nervous system in some ASD subjects.

Since the observed changes in miRNA expression indicate that multiple steps of the signaling pathways may be affected, including other inflammatory/regulatory cytokines, we also examined whether levels cytokines other than IL-1β and IL-10 were also changed in association with IL-1ß/IL10 ratios. Indeed, we observed low production of TNF-α, an inflammatory cytokine, in ASD PBMo with low IL-1ß/IL-10 ratios and also low production of TGF-ß, a counter-regulatory cytokine, in ASD PBMo with high IL-1ß/IL-10 ratios. These results correlate to the changes in miRNA expression in ASD PBMo, given their functions as explained in the previous paragraph. Upregulation of miR-181a in ASD PBMo with high IL-1ß/IL-10 ratios may indicate that miR-181a was upregulated to counter-regulate excessive activation of the PI3K/Akt pathway, which may result in over-activation of monocytes.

In this study, we also examined whether ASD behavioral symptoms change in association with changes in cytokine production profiles in ASD PBMo. When the data were examined, on the basis of IL-1ß/IL-10 ratios, we found associations between cytokine levels and ASD behavior scores do change. Namely, ABC subscale II (lethargy) scores were lower when ASD PBMo revealed low IL-1ß/IL-10 ratios (Table 5), which may reflect suppression of inflammation. ABC subscale V (inappropriate approach) scores were lower when ASD PBMo revealed high IL-1ß/IL-10 ratios (Table 5), which may reflect decrease in spontaneous speech from our clinical impression.

Interestingly, ABC irritability scores were negatively associated with levels of inflammatory cytokines (IL-1ß, IL-6) under cultures with stimulants, but positively associated with another inflammatory cytokine, TNF-α (Table 6). Such associations were less evident when ASD PBMo revealed high or normal IL-1ß/IL-10 ratios (Table 6). We observed a similar trend in ABC subscale IV (hyperactivity) (data not shown). In contrast, when ASD PBMo revealed high IL-1ß/IL-10 ratios, ABC scores assessed at the same time as PBMo sample obtainment revealed a positive association between IL-6 and IL-1ß levels and ABC subscale III (stereotypy) (Table 7). We did not observe a close association in changes in ABC scores with TGF-ß or IL-10 levels produced by PBMo in this study (Tables 6 and 7 and data not shown). These results may be interpreted that when innate immune responses are deviated from normal homeostasis, such aberrant responses significantly affect ASD behavioral symptoms, while when responses are not deviated, ASD core behavioral symptoms are not affected. However, further study with careful mechanistic evaluation will be necessary to assess our initial interpretation, in addition to the validation of the data with a larger number of study samples.

We also studied whether other clinical features and co-morbid conditions were associated with changes in IL-1ß/IL-10 ratios (Table 8). Since the same subjects can reveal either high or low IL-1ß/IL-10 ratios, depending on the time of sample obtainment [25], frequencies of co-morbid conditions and cognitive activity/adaptive skills were compared in ASD subjects with high/low ratios vs. those with normal ratios. ASD subjects with high/low ratios revealed lower adaptive skills and a higher frequency of non-IgE-mediated food allergy. We also observed a tendency for higher frequency of both seizure disorders and specific antibody deficiency, as observed in our previous study, which had a larger number of study subjects, although we did not examine miRNA expression in that study [25].

## Conclusions

Our results indicate that changes in miRNA expression and cytokine production profiles are associated with changes in ASD behavioral symptoms, as well as frequency of co-morbid conditions in a subset of ASD subjects. The changes in miRNA expression that we detected along with changes in cytokine levels by ASD PBMo indicate that these changes may cause dysregulation in PTEN-mediated signaling pathways. This may in turn affect ASD behavioral symptoms. Our findings also support a role of immune-mediated inflammation in ASD and feasibility of the use of immune-modulating agents in ASD subjects with evidence of immune-mediated inflammation. Additional studies will be helpful to further assess the utility of miRNA expression and IL-1ß/IL-10 ratios by PBMo, as biomarkers of immune-mediated inflammation in ASD. Given the secretary natures of monocytes, serum miRNA levels may also serve as biomarkers of immune-mediated inflammation, as mir-223 being reported to be a biomarker for an inflammatory bowel disease [50].

## Additional files

Additional file 1: A complete list of miRNAs showing differential expression (≥ 2 fold) between groups shown in Table 2. (XLSX 11 kb)

Additional file 2: Results of *Z* test of miRNA expression among ASD groups and non-ASD controls cells. (XLSX 182 kb)

Additional file 3: Results of *Z* test of miRNA expression between whole ASD cells and non-ASD control cells. (XLSX 261 kb)

Additional file 4: Results of miRNA target gene analysis of group A. (XLSX 626 kb)

Additional file 5: Results of miRNA target gene analysis of group B. (XLSX 543 kb)

Additional file 6: Results of miRNA target gene analysis of group C. (XLSX 105 kb)

## Abbreviations

ABC: Aberrant Behavior Checklist
AC: Allergic conjunctivitis
ADI-R: Autism Diagnostic Inventory-Revisited
ADOS: Autism Diagnostic Observational Scale
Akt: Protein kinase B activated through PI3K-Akt pathway
AR: Allergic rhinitis
ASD: Autism spectrum disorder
IL: Interleukin
MIA: Maternal immune activation
NFA: Non-IgE-mediated food allergy
PB: Peripheral blood
PBMo: Peripheral blood monocytes
PI3K: Phosphoinositide 3-kinase
PTEN: Phosphatase and tension homolog
PST: Prick skin testing
SPUH: Saint Peter’s University Hospital
TGF: Transforming growth factor
TLR: Toll-like receptor
TNF: Tumor necrosis factor
VABS: Vineland Adaptive Behavioral Scale
